# Results of a pilot study in the U.S. and Vietnam to assess the utility and acceptability of a multi-level pregnancy test (MLPT) for home monitoring of hCG trends after assisted reproduction

**DOI:** 10.1186/s12905-017-0422-y

**Published:** 2017-08-22

**Authors:** Tara Shochet, Ioanna A. Comstock, Nguyen Thi Nhu Ngoc, Lynn M. Westphal, Wendy R. Sheldon, Ly Thai Loc, Jennifer Blum, Beverly Winikoff, Paul D. Blumenthal

**Affiliations:** 1 Gynuity Health Projects, 15 East 26th Street, Suite 801, New York, NY 10010 USA; 2 George Washington University, 2150 Pennsylvania Ave NW, Washington, DC 20036 USA; 3 Center for Research and Consultancy in Reproductive Health, 16D, Luy Ban Bich, Tan Thoi Hoa, Tan Phu District, Ho Chi Minh City, 70000 Vietnam; 4 Reproductive Endocrinology, Stanford University, 900 Welch Road, Suite 20, Palo Alto, CA 94304 USA; 5 Hung Vuong Hospital, 128 Hong Bang Street, Ward 12, District 5, Ho Chi Minh City, Vietnam; 6 Stanford University Medical Center, 300 Pasteur Drive, Stanford, CA 94304 USA

**Keywords:** Multi-level pregnancy test, Semi-quantitative pregnancy test, Assisted fertility, hCG

## Abstract

**Background:**

To evaluate the utility and acceptability of using multi-level pregnancy tests (MLPTs) at home to monitor hCG trends following assisted reproductive technology (ART).

**Methods:**

One hundred and four women presenting for ART at either Stanford Medicine Fertility and Reproductive Health Clinic (Stanford, CA) or Hung Vuong Hospital (Ho Chi Minh City, Vietnam) participated in this pilot study. Women were asked to perform the MLPT at home, primarily on days when they were also scheduled to receive standard clinic-based serum hCG testing. These tests were administered up to 6 times over the 6-week period following embryo transfer or intrauterine insemination (IUI). Concordance of serial hCG readings for each time point was assessed by comparing trends in urine MLPT results with trends in serum hCG. Stable or increasing hCG level was interpreted as an indication of a progressing pregnancy, while a declining hCG was interpreted as a lack of established or progressing pregnancy. At study end, all participants were asked about the acceptability and convenience of using the MLPT at home for monitoring hCG trends following ART.

**Results:**

Data from both urine and serum testing are available for 156 of 179 clinic visits (87.2%). There was high concordance of serial trend results between the two types of tests: among the 156 sets of serum and urine hCG data points, 150 (96.2%) showed a matching trend in hCG pattern and 6 (3.8%) resulted in a discordant trend. Seventy-three percent of women reported being satisfied or very satisfied with using the MLPTs at home. Almost all (96.6%) said that the MLPT was easy or very easy to use.

**Conclusion:**

The MLPT offers women and health care providers a client-friendly diagnostic tool to detect very early pregnancy and monitor its progress.

**Trial registration:**

This study was registered on clinicaltrials.gov as NCT01846403 (May 1, 2013), and NCT01919502 (August 5, 2013).

## Background

Approximately 1.5 million assisted reproductive technology (ART) cycles are performed each year worldwide [1]. In addition to payments for medications, serial ultrasounds, hormonal assays, and other laboratory exams, there are the costs to monitoring ART outcomes. Women are expected to comply with numerous clinic visits throughout the process, resulting in time away from employment and/or home [2]. Standard monitoring protocols after embryo transfer include sequential serum hCG analyses and ultrasound – all done on a regular (at times weekly) basis in a clinic or lab setting. However, ultrasound generally does not show the presence of a gestational sac until 4 weeks after embryo transfer, and repeat ultrasounds may be necessary to confirm a continuing pregnancy. Serum hCG requires a blood draw and lab testing. In the best of circumstances, women receive their lab results the afternoon of their serum blood test. In any event, women have already left the clinic before the result is known.

To complement existing tools, we tested repeat use of a multi-level pregnancy test (MLPT) as an outpatient method for women to self-monitor ART outcomes. The test can be performed serially to ascertain initial presence of the pregnancy hormone hCG and to monitor changes in hCG levels in the early weeks after embryo transfer or intrauterine insemination (IUI). Thus a stable or increasing hCG level would indicate a progressing pregnancy, while a negative or decline in hCG would indicate otherwise. To date, these tests have primarily been used in research that focuses on their role in assessing ongoing pregnancy after medical abortion [3–6]. At-home use of an MLPT could allow women to monitor their hCG progress more frequently than current clinic-based monitoring protocols, as well as more quickly, as the test provides results in 15 min.

Previous research assessing medical abortion outcomes compared an MLPT to serum hCG; the sensitivity and specificity of the urine test in determining a serum level above 1000 mIU/mL were 88.6% and 71.7%, respectively [7]. Additional research has shown that serial use of MLPTs is an effective means for ascertaining early medical abortion outcomes by monitoring hCG levels pre- and post- medical abortion [3–6]. In these studies, the trend in hCG range was used to assess the possibility of ongoing pregnancy with a decline in hCG range suggesting no ongoing pregnancy and a stable or increase in hCG range suggesting the need for further evaluation. Extrapolating from this use, we theorized that MLPT trend data could also be used to assess pregnancy progress.

Our pilot study sought to evaluate the concordance between MLPT and serum hCG trend data following ART, and to examine the acceptability of performing repeat multi-level pregnancy tests to monitor hCG levels at home. If successful, it could serve as an at-home test to help women monitor the success of their assisted fertility treatment.

## Methods

We conducted a pilot study at Stanford Medicine Fertility and Reproductive Health Clinic (Stanford, CA) and at the infertility clinic of Hung Vuong Hospital (Ho Chi Minh City, Vietnam). Women presenting for in vitro fertilization (IVF) or intrauterine insemination (IUI) at Stanford, or for IVF at Hung Vuong, were invited to participate. Inclusion criteria were being eligible for the assisted fertility treatment per clinic guidelines, agreeing to a series of blood draws for serum hCG testing, agreeing to return for a series of follow-up visits, willingness to follow provider and written instructions regarding use of the at-home pregnancy test, and willingness to provide contact information for follow-up. Participants also needed to be able to read and write in English (U.S.) or in Vietnamese (Vietnam). IRB approval was granted by Stanford’s Institutional Review Board and Hung Vuong Hospital’s Ethical and Scientific Committee. All participants provided written informed consent.

**Fig. 1:**
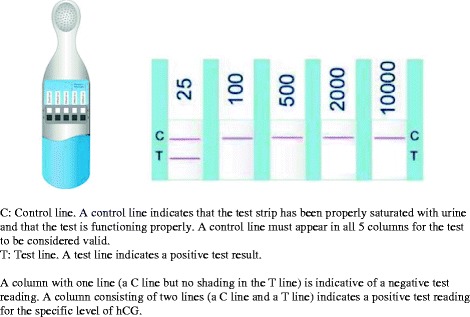
The dBest® multi-level pregnancy test with reading of at least 25 mIU/mL of hCG

Following enrollment, each participant was given an MLPT, a urine collection cup, a home diary card, and written and verbal instructions illustrating the proper use of the MLPT. Women were also reminded that this was a pilot study and that they should rely on their serum results to guide their care. The tests used at the two study sites were identical but had different brand names: the Quanti5® (Athenium Pharmaceuticals, USA) at Stanford and dBest® (AmeriTek Inc., USA; see Fig. 1) at Hung Vuong. Both tests read hCG levels in one of five ranges: 25-99, 100-499, 500-1999, 2000- 9999, and ≥10,000 mIU/ml. Women documented the test result at home by circling a picture that matched their result and then completed a short questionnaire to interpret the result and provide feedback on acceptability. They returned the completed home card at the next clinical appointment. Women were provided additional MLPTs as needed at the end of each appointment. At study end, clinic staff asked each participant a series of 15 brief questions regarding the acceptability and convenience of using the MLPT as a part of fertility treatment follow-up. These interviews were conducted in person in Vietnam, and by phone in the U.S. Women were told in the informed consent that the exit interview would take no longer than 30 min.

Protocols regarding the timing and frequency of the testing differed due to differences in standard clinic practice at the two sites:

### Stanford Medicine Fertility and Reproductive Health Clinic

Women were instructed to use their first MLPT exactly 14 days after an intrauterine insemination on the same day as their routine serum beta hCG. If a woman had a positive beta hCG, she was asked to complete another MLPT and home diary card 48 h later when a repeat beta hCG was routinely performed. If a negative serum pregnancy test was shown, participation in the study was recorded as complete with no further follow-up necessary aside from the exit interview.

All other participants were instructed to complete another MLPT and home diary card on the morning of their dating ultrasound (approximately 6.5 weeks gestation) to monitor the progress of their pregnancies. The physician decided the most appropriate follow-up for each woman depending on the findings of the transvaginal ultrasound. Study participation for pregnant women ended after the final obstetrical ultrasound in the infertility clinic.

### Hung Vuong Hospital’s infertility clinic

Participants were asked to administer an MLPT at home up to 6 times (every 7 days starting at day 7 post-transfer) over the 6-week period following embryo transfer. In addition, participants were asked to return to the hospital for clinical assessment, which included both serum hCG and ultrasound, approximately 14, 21, and 28 days and at 6 weeks post-embryo transfer. On two occasions (7 days and 5 weeks post-transfer) women were asked to perform an MLPT at home, although there was not a corresponding clinic visit on this day. On days when a hospital visit was scheduled, women were asked to perform the MLPT at home prior to the appointment. Participants were instructed to call their provider if any of the MLPTs performed at home on days when a hospital visit was not scheduled showed a decrease in hCG, signified by a drop in bracketed range displayed on the MLPT; a clinic visit was then scheduled. If an MLPT showed no change or increased, women proceeded as planned with their next scheduled hospital visit. Midway through the pilot, the procedures were modified to allow for all MLPTs scheduled for clinic appointment days to be performed at the clinic just prior to each participant’s blood draw. Women were discharged from the study if not pregnant by day 14 post-transfer or ended before the 6 week visit.

The primary aim of the pilot study was to document whether continuing pregnancy could be initially identified and then monitored at home using a multi-level pregnancy test. Measures included concordance of MLPT and serum hCG trend data from tests conducted on the same day, and acceptability of using an MLPT at home. As this was a pilot study, we selected a minimum sample size of 50 women per site. We wanted to enroll a reasonable number of women to begin to understand if MLPTs could be an effective monitoring tool in an assisted fertility service and to guide future research. We produced descriptive results from the characteristic and acceptability data, and conducted a concordance analysis with kappa statistic using the MLPT and serum test findings. To evaluate concordance, we utilized the circled MLPT results and determined increasing/decreasing/stable hCG independent of the women’s interpretations by comparing the result with the previous test. For the first test, we compared to baseline hCG, which we assumed to be 0 for all participants. Data were entered into SPSS version 19.0 (IBM, Armonk, NY, USA), and all analyses were conducted using STATA versions 11 & 12.1 (StataCorp, College Station, TX, USA).

## Results

Fifty women were enrolled at Hung Vuong between June and December 2013, and 54 women at Stanford between October 2013 and March 2014. Participant characteristics for both studies are presented in Table 1. During the study, 42 women (40.4%) exhibited signs of pregnancy including an increase in hCG (≥ 25 mIU/ml) as measured by either MLPT or serum test. Of these, 35 (83.3%) were pregnant at study end. One additional pregnant woman in Vietnam dropped out during the study due to personal concerns regarding threatened abortion. Three women in the U.S. were lost to follow-up before any study data were collected. Only three women in the U.S. had both MLPT and ultrasound done on the same day, which inhibited us from comparing urine hCG and ultrasound readings.

**Table 1:** Participant characteristics: median (range) or % (n)

	*n* = 104
Age in years	34 (22–45)
Education completed	
Primary school	4.8 (5/104)
Secondary school	22.1 (23/104)
University or higher	73.1 (76/104)
History of pregnancy	44.2 (46/104)
History of live birth	23.8 (24/101)
Pregnant at study end	33.7 (35/104)

Data from both urine and serum testing are available for 156 of 179 clinic visits (87.2%). Data were missing or incomplete for 14 MLPTs and 3 serum tests; 3 MLPTs were reported to have had an inconclusive result; 2 women declined to have a serum test following two sequential negative MLPTs; and 1 woman declined the serum test as she began menstruating prior to the blood draw.

There was high concordance of serial trend results between the two types of tests (Table 2): among the 156 sets of serum and urine hCG data points, 150 (96.2%) showed a matching trend in hCG pattern and 6 (3.8%) resulted in a discordant trend (kappa = 0.91 (95% CI: 0.84–0.98)). There were no clear patterns in terms of characteristics of women with a discordant result at any time point.

**Table 2:** Concordance between serum and MLPT results^a^: % (n)

	Serum showed steady or increase in hCG^b^ and at least 25 mIU/ml	Serum showed hCG < 25 or decrease in hCG^b^
MLPT showed steady or increase in hCG^b^ and at least 25 mIU/ml (*n* = 106)	99.1 (105)	0.9 (1)
MLPT showed hCG < 25 or decrease in hCG^b^ (*n* = 50)	10.0 (5)	90.0 (45)

^a^There were 6 MLPTs with no serum test with which to compare,14 serum tests with no MLPT with which to compare, and 3 inconclusive MLPTs

^b^As compared to previous test. For the first test, we compared to an assumed 0 hCG level pre-ART

Kappa = 0.91 (95% CI: 0.84–0.98)

Women’s interpretation data were available for 238 MLPT data points (includes data from additional home tests taken by women in Vietnam on days in which there was no clinic visit). Of the tests that showed a negative result or decrease in hCG (*n* = 108), the woman interpreted the result to indicate she was not pregnant two-thirds of the time (66.7%, *n* = 72/108) and that she was pregnant 2.8% of the time (*n* = 3/108). Women reported being unsure 30.6 % of the time (*n* = 33/108). Of the tests that showed a positive result (*n* = 130), 91.5% (*n* = 119/130) were correctly reported as indicating a positive pregnancy; 9 (6.9%) indicated uncertainty, and 2 (1.5%) incorrectly interpreted the result as meaning not pregnant.

Satisfaction with using the MLPTs for at-home hCG monitoring was high, with 73.6% of women reporting being satisfied or very satisfied (Table 3). Only four women said that they were unsatisfied. Of the six women who had discordant serum and urine hCG results at one point in the study, four reported that they were satisfied or very satisfied with the process (data not shown).

**Table 3:** Acceptability of using MLPTs for at-home monitoring: %(n)

	*n* = 87
Satisfaction with using MLPTs to monitor pregnancy progress	
Very satisfied/satisfied	73.6 (64/87)
Neither satisfied nor unsatisfied	21.8 (19/87)
Unsatisfied	4.6 (4/87)
Ease of using MLPT	
Very easy/easy	96.6 (84/87)
Neither easy nor difficult	3.4 (3/87)
Felt very confident/confident in MLPT’s ability to help monitor pregnancy progress	86.0 (74/86)
Having additional information from the MLPTs made woman feel…	
More relaxed about the IVF procedure	52.3 (45/86)
No difference	25.6 (22/86)
Less relaxed about the IVF procedure	22.1 (19/86)
Would want to use home pregnancy tests to monitor pregnancy progress if had fertility treatment again in future	76.7 (66/86)
Would recommend using home pregnancy tests in addition to hospital visits to a friend having fertility treatments	84.9 (73/86)
Using home MLPTs in lieu of some hospital visits would be more convenient/save time	76.7 (66/86)

The vast majority of women (96.6%) said that the MLPT was easy or very easy to use, and that they felt confident or very confident in the MLPT’s ability to help monitor pregnancy progress (86.0%). Approximately three-fourths of participants (76.7%) would want to use MLPTs again to monitor pregnancy progress if they had future fertility treatments, and 85% would recommend using MLPTs to a friend undergoing ART.

## Discussion

HCG trend data based on MLPT results corresponded very well with those based on serum lab results. This finding is not surprising based on previous research demonstrating a correlation between urine and serum measures of hCG [7]. The results also provide further evidence that women can successfully use the MLPT and find it highly acceptable as a reproductive health tool, as has been shown in several previous studies for medical abortion [3–6]. These data offer an early indication that an MLPT can provide an accurate assessment of hCG trends for women undergoing ART and may be a practical at-home monitoring instrument.

The ability to determine hCG range easily, quickly, and outside of the clinic setting, could result in improved quality of care for women undergoing fertility treatment. Quality of care research with women undergoing IVF found that accessing information following treatment was one of the top two most important aspects of care [8]. At-home use of the MLPT could allow women to increase the frequency and the ease of accessing data regarding their potential pregnancy. Further research could establish a role for the MLPT as a supplement to clinic-based serum lab tests, potentially reducing costs and time burdens for both women and health care systems [9].

Participants in Vietnam performed the initial MLPT at home on Day 7 post-embryo transfer. At this time point, 5 (20%) of the 25 women who became pregnant during the study had a positive result on the MLPT. From this, we suggest that women could be counseled that, although the sensitivity is low, it is possible to show a positive result using the MLPT as early as day 7.

In Vietnam, there were some concerns mid-way through the study about whether or not women were waiting long enough before reading their test results and study staff decided to implement a procedure modification whereby women would administer the test at the clinic and be sure to wait the full 15 min before reading the results. We reviewed the data both before and after this procedural change was made and found that the trends shown in MLPT ranges almost always matched the trends in serum hCG both before and after the modification. The discordant readings in both studies mostly occurred at the lower end of the MLPT ranges, suggesting that there may be some imprecision around the boundaries at these lower values.

This pilot study represents the first time to our knowledge that an MLPT was used as part of ART care in a clinical study. The sample was small in each country and we are not able to make any generalizations about use for this indication. As ART is very woman-dependent, as well as clinic- and clinician-dependent, we were not able to develop a single protocol for the two sites. In spite of these challenges, we were able to collect both urine pregnancy test data and serum hCG data from the majority of participants. If additional research shows the MLTP to indeed be of use to women using ART, creating guidelines for standardized use might pose a similar challenge.

Given its concordance with serum hCG trends, as well as its high acceptability among participants, the MLPT could be a valuable addition to standard ART care that could be used at home as a supplement to current protocols. In fact, many participants reported that it would be convenient to substitute some of the scheduled hospital visits and blood draws with MLPTs, although it is possible that this was influenced by the extra visit and blood draws added for study purposes. In addition, in some low-resource settings, serum hCG tests may be less available than in high-resource settings and an MLPT with known performance characteristics and fixed, standardized brackets might work similarly to serial serum testing in demonstrating hCG trends. However, there is very limited commercial accessibility to the MLPT. Without product availability, little movement can be made towards integrating MLPTs into standard ART.

## Conclusions

The MLPT offers women and health care providers a client-friendly diagnostic tool to detect early pregnancy and monitor its progress. Use of such a test at home might improve the quality and convenience of care for women.

## Abbreviations

ART: assisted reproductive technologies
hCG: human chorionic gonadotropin
IUI: intrauterine insemination
IVF: in vitro fertilization
MLPT: multi-level pregnancy test
